# Doing More With Less: A Multitask Deep Learning Approach in Plant Phenotyping

**DOI:** 10.3389/fpls.2020.00141

**Published:** 2020-02-28

**Authors:** Andrei Dobrescu, Mario Valerio Giuffrida, Sotirios A. Tsaftaris

**Affiliations:** ^1^ IDCOM, University of Edinburgh, Edinburgh, United Kingdom; ^2^ School of Computing, Edinburgh Napier University, Edinburgh, United Kingdom

**Keywords:** plant phenotyping, deep learning, multitask, leaf count, PLA, genotype

## Abstract

Image-based plant phenotyping has been steadily growing and this has steeply increased the need for more efficient image analysis techniques capable of evaluating multiple plant traits. Deep learning has shown its potential in a multitude of visual tasks in plant phenotyping, such as segmentation and counting. Here, we show how different phenotyping traits can be extracted simultaneously from plant images, using multitask learning (MTL). MTL leverages information contained in the training images of related tasks to improve overall generalization and learns models with fewer labels. We present a multitask deep learning framework for plant phenotyping, able to infer three traits simultaneously: (i) leaf count, (ii) projected leaf area (PLA), and (iii) genotype classification. We adopted a modified pretrained ResNet50 as a feature extractor, trained end-to-end to predict multiple traits. We also leverage MTL to show that through learning from more easily obtainable annotations (such as PLA and genotype) we can predict a better leaf count (harder to obtain annotation). We evaluate our findings on several publicly available datasets of top-view images of *Arabidopsis thaliana*. Experimental results show that the proposed MTL method improves the leaf count mean squared error (MSE) by more than 40%, compared to a single task network on the same dataset. We also show that our MTL framework can be trained with up to 75% fewer leaf count annotations without significantly impacting performance, whereas a single task model shows a steady decline when fewer annotations are available. Code available at https://github.com/andobrescu/Multi_task_plant_phenotyping.

## Introduction

Nondestructive, image-based plant phenotyping is a growing trend in how scientists and breeders engage in plant characterization. Due to the advances in image acquisition systems (Qiu et al., 2018) and development of affordable hardware and software framework (Dobrescu et al., 2017b; Minervini et al., 2017), high throughput plant image capture is becoming widespread. In particular, machine learning has shown that it can be applied effectively in processing vasts amounts of data, including in plant phenotyping problems (Scharr et al., 2016). For example, segmenting whole plants (Minervini et al., 2014; Aich and Stavness, 2017), or each individual leaf (Romera-Paredes and Torr, 2016; Ren and Zemel, 2017; Ward et al., 2018), synthetic image synthesis (Giuffrida et al., 2017; Zhu et al., 2018), and leaf counting (Aich and Stavness, 2017; Dobrescu et al., 2017a; Giuffrida et al., 2015; Pape and Klukas, 2015; Giuffrida et al., 2018b; Itzhaky et al., 2018) are all phenotyping tasks that have been recently addressed using machine learning and deep learning, technologies that are becoming more common in the plant-research community. In fact, the fourth edition of the *Computer Vision Problems in Plant Phenotyping* ^1^ workshop (CVPPP 2019) shows an increasing interest from people inside and outside the plant phenotyping community to invest efforts to develop newer machine learning based approaches.

Leaf count has been an area of interest for plant phenotyping, as it is related to developmental stages (Boyes et al., 2001) and can be an indicator for yield potential (Ngouajio et al., 1999) and plant health (Rahnemoonfar and Sheppard, 2017). Two have been proposed to infer leaf count: (i) determining the leaf count as a subproduct of per-leaf segmentation; or (ii) tackling the problem as a holistic regression task. Several different algorithms have been proposed for a per-leaf segmentation approach. Scharr et al. (2016) discusses four methods to achieve per-leaf segmentation, where machine learning was used for the first time for this task. Romera-Paredes and Torr (2016); Ren and Zemel (2017); Ward et al. (2018), and Zhu et al. (2018) have proposed several deep learning approaches for per-leaf segmentation, obtaining remarkable results in terms of segmentation accuracy. However, the main issue with such methods is that they require per-leaf segmentations to train the algorithms that are often time-consuming, laborious, and expensive to acquire. Although Minervini et al. (2015; 2017) have proposed semiautomatic graphical tools, they still require experienced users to obtain an adequate per-leaf segmentation. Another type of annotation used for leaf counting is to mark each leaf with a dot on the center, rather than the whole leaf segmentation. Although it is an easier way to provide topological and localisation information, it still requires a human to click on the center of each leaf. Itzhaky et al. (2018) use such annotation to train a leaf detector which is used in conjunction with a leaf regressor (named D+R) to achieve state-of-the-art leaf count.

Alternatively, leaf counting can be addressed as a holistic regression task, where an algorithm predicts the total leaf count in an image. In this context, the machine learning algorithm requires just the total number of leaves, which is an easier annotation to obtain, compared to the per-leaf segmentations (Minervini et al., 2015; Giuffrida et al., 2018a). The first studies to use machine learning techniques reported encouraging results (Giuffrida et al., 2015; Pape and Klukas, 2015), although more recently approaches based on deep neural networks have become the state of the art. Dobrescu et al. (2017a) proposed a deep neural network based on a ResNet50 (He et al., 2016), where leaf counting was learned by agglomerating data from multiple sources. Further to this, Giuffrida et al. (2018b) proposed a versatile network that demonstrated that leaf counting could be better learned using data from multiple imaging modalities using a single architecture. Itzhaky et al. (2018) also describe another approach (named MSR) which uses a feature pyramid network architecture (Lin et al., 2017) to learn a direct regressor at multiple scale levels of a plant and then fuse them to output a single leaf count prediction. Ubbens and Stavness (2017) proposed several specialised deep network architectures to count leaves in different datasets, as well as to infer other tasks such as projected leaf area (PLA) and genotype prediction.

The success of machine learning, and especially deep learning, is attributed to the ability to relate images to a given task. Deep neural networks extract meaningful information from images (typically referred to as *image features*), even when they contain complex structures like plants. In the current paradigm, many machine learning models are specialised to perform a single task (i.e., learn one plant trait at a time).

However, plant phenotyping traits, such as the total leaf count, can often be related to other traits, such as the total leaf area, age, and genotype. Incorporating such related traits in the deep learning framework would help the deep neural network better learn all the traits (Caruana, 1997).

Multitask learning (MTL) has been shown to improve the accuracy and the generalization performance of each task (Caruana, 1997). The benefits of MTL are multifold, especially when tasks are related to each other. Firstly, one one network is trained to perform multiple tasks at the same time, in contrast with Ubbens and Stavness (2017), where several networks with different architectures were trained separately to extract phenotyping traits. The learning of multiple tasks enforces the network to learn good representations, thus increasing the generalization capability of the model. Since information sharing is the core of MTL, learning multiple tasks simultaneously reduces overfitting, even in presence of reduced datasets (Baxter, 1997). Additionally, from an implementation perspective, MTL allows having just one shared model instead of independent models per task. This helps reduce storage space, decreases training times and is easier to deploy and maintain. MTL is a special case of transfer learning (Pan and Yang, 2010), where (i) there is no distinction between tasks; and (ii) the objective is to increase performance for all the involved tasks.

Surprisingly, despite the benefits of MTL and its application in several other areas of computer vision (Ramsundar et al., 2015; Kokkinos, 2017; Ranjan et al., 2019), it has been under-explored in addressing problems in plant phenotyping. Pound et al. (2017) proposed the earliest application in MTL for plant phenotyping, where a deep neural network that can both detect and count wheat spikes, as well as classify the presence of awns.

**Figure 1:**
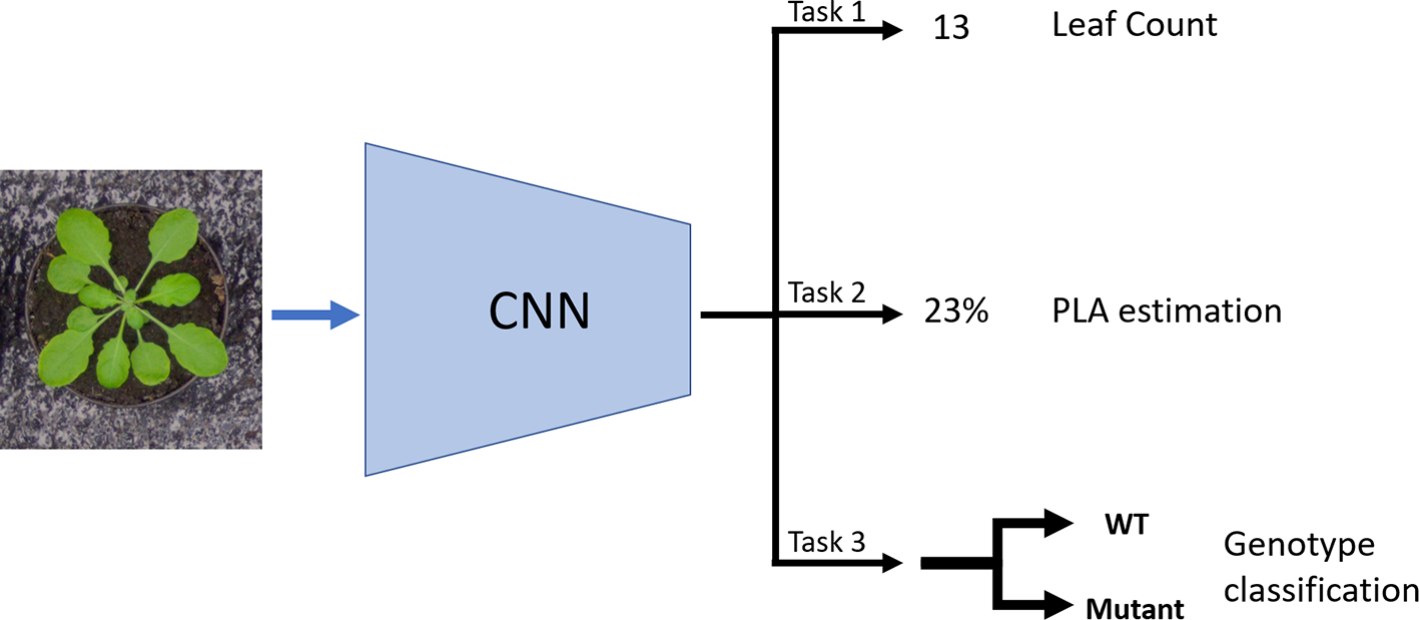
Schematic of the multitask learning (MTL) model: The model takes in an image as input and it uses a CNN to learn three tasks concurrently: Leaf count outputted as a scalar (Task 1); projected leaf area (PLA) estimation as a percentage of area the plant occupies in the image (Task 2); and genotype classification between mutant and wild type (Task 3).

In this paper, we propose an MTL architecture aimed to infer leaf counting, together with the PLA and genotype classification (**Figure 1**). We use the dataset *Ara2013* (Minervini et al., 2017) and show that multiple tasks help to achieve more precise predictions of these three plant traits. The tasks were chosen, as they are relevant and well known plant phenotyping objectives as well as being correlated to each other, which helps the training process. The PLA and genotype annotations are less tedious and time-consuming to gather. The PLA can be obtained with a plant segmentation algorithm (Aich and Stavness, 2017; Dobrescu et al., 2017a; Minervini et al., 2015), whereas the genotype is generally known *a priori* to the scientists. The leaf counting task and PLA estimation are treated as direct regression problems having only the total leaf count and total PLA as respective annotations. The genotyping task is addressed as a binary classification between wild-type and mutant.

The contributions of this paper are multifold:

- Our end-to-end MTL architecture predicts several traits at the same time, in particular leaf counting, PLA, and genotype. Having one unified model for multiple tasks improves performance in leaf count compared to a single task model. Amongt the other tasks, leaf counting is the hardest to predict from a computer vision perspective, due to huge variability between leaves as well as occlusions in the images.
- We show that our proposed method can be trained with fewer leaf count annotations without significantly impacting leaf count performance. Our results show that when annotations for one task are available, performance can be improved by using correlated tasks for the same images.
- We show which count annotations have the most impact on the model performance. Understanding this key aspect would help guide the annotation of a new dataset, highlighting which images should be first annotated in a new dataset.

## Methods

For this study we developed an MTL deep learning model that takes in as input a top-down color (RGB) image of a rosette plant (e.g. *Arabidopsis thaliana*) to infer the total number of leaves, PLA, and binary genotype classification.

### The Feature Extractor

**Figure 2:**
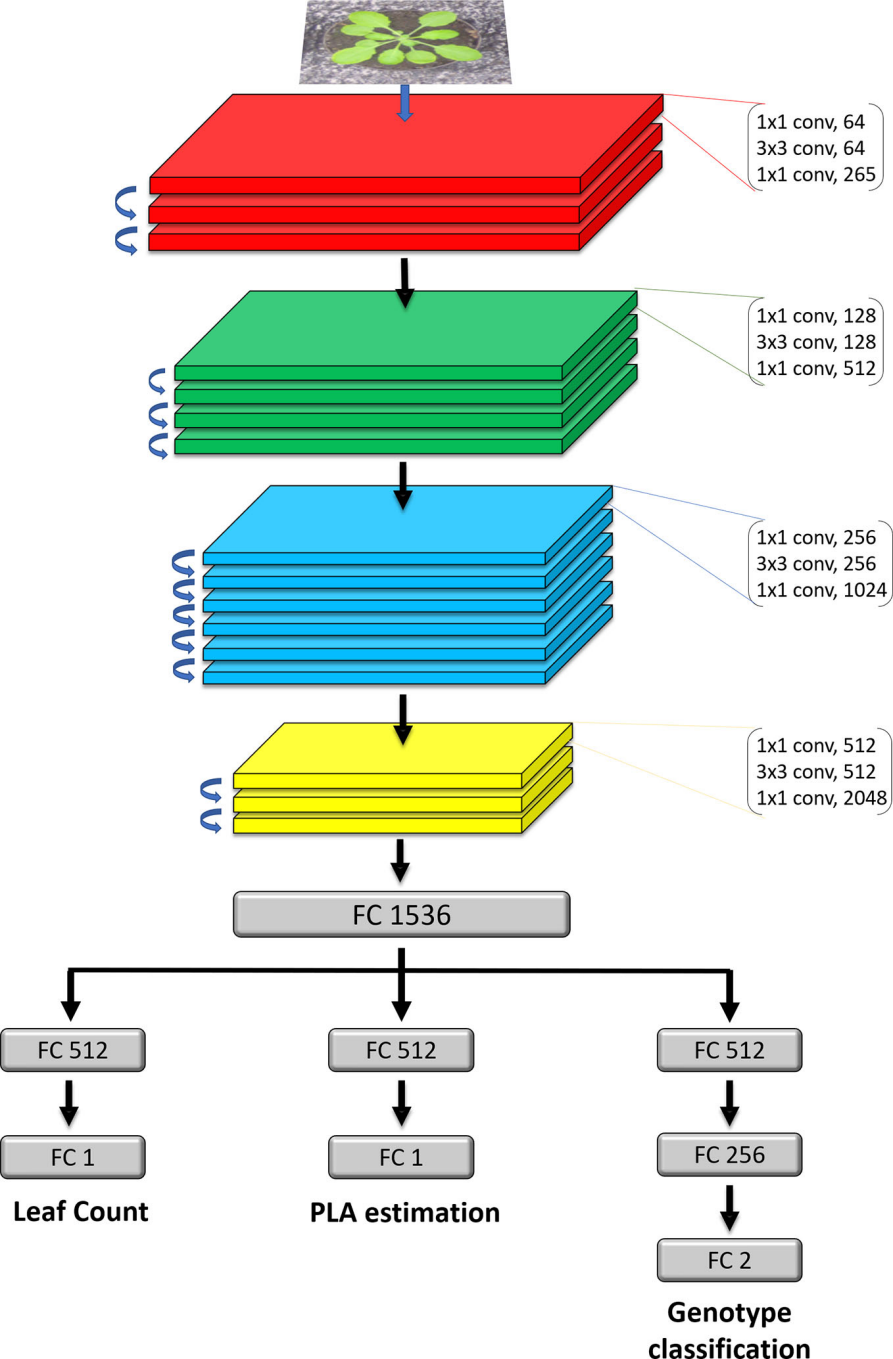
Detailed architecture of the model. The network takes in as input an RGB image of a rosette plant. The main feature extractor is a ResNet50 deep residual network, which is composed of 16 residual blocks which consist of three stacked layers with residual connections between the input and the output of each block. FC layers represent fully connected layers of a given size. The FC1536 is a shared dense representation layer from which each task branch off into their respective output. Each branch is then is specialised for a specific task.

The first part of the model (**Figure 2** Top) is a ResNet50 (He et al., 2016) neural network and works as a feature extractor. We used a ResNet50 pretrained on ImageNet (Krizhevsky et al., 2012), as it has been demonstrated to perform well on plant phenotyping tasks (Dobrescu et al., 2017a; Giuffrida et al., 2018b). The architecture of the model is composed of 16 convolutional blocks, each consisting of three convolutional layers of increasing filter sizes to maintain complexity per layer (He et al., 2016). This model is a residual neural network, which means that the convolutional layers are not just stacked on top of each other, but also additional connections between the convolutional blocks (residual connections) are present between neighboring blocks. These skip connections help propagate the error signal faster across these very deep networks layers, yielding improved results over other network designs. We modified the reference ResNet50, by removing the last layer intended for classification and replaced it with a fully connected layer containing 1536 nodes, which acts as a shared representation for the three training tasks. Up to and including the shared representation, we leverage hard parameter sharing, meaning the network layers are shared between all the tasks. This approach reduces the risk of overfitting which is important when training deep learning models.

### The Task Branches

The second part of the model (**Figure 2** Bottom) consists of the three task-specific branches that are each responsible for computing one of the tasks. The branches receive information from the shared representation above and specialise on one task. The first one computes the leaf count and it consists of a fully connected layer of 512 nodes and a 1 node layer which outputs the count prediction. The second for estimating the PLA, has the same design as the leaf count branch. The PLA output is normalised as the percentage that the plant occupies in relation to the whole image. Genotype classification is determined by the third branch and contains 3 fully connected layers of 512, 256 and 2 nodes respectively. The activation functions of the fully connected layers in the branches are rectified linear units (ReLU), except for the final genotype and PLA prediction layers, which are sigmoid and LeakyReLU respectively. On layers before the final prediction layers for all three tasks we apply an L2 regularization of 0.04 to penalize layer activity during training and prevent overfitting.

### Losses

All tasks are learned at the same time in the MTL model. Each task has a specific loss tailored to the specifications. For the leaf counting and PLA estimation tasks the loss is mean squared error (MSE). However, when comparing to the other tasks the values were very low. To balance it out, we multiplied the error values by 10 to maintain comparable values. For the task of genotype prediction the loss is binary cross entropy using a sigmoid final layer activation to get the output between 0 and 1.

### Datasets

**Figure 3:**
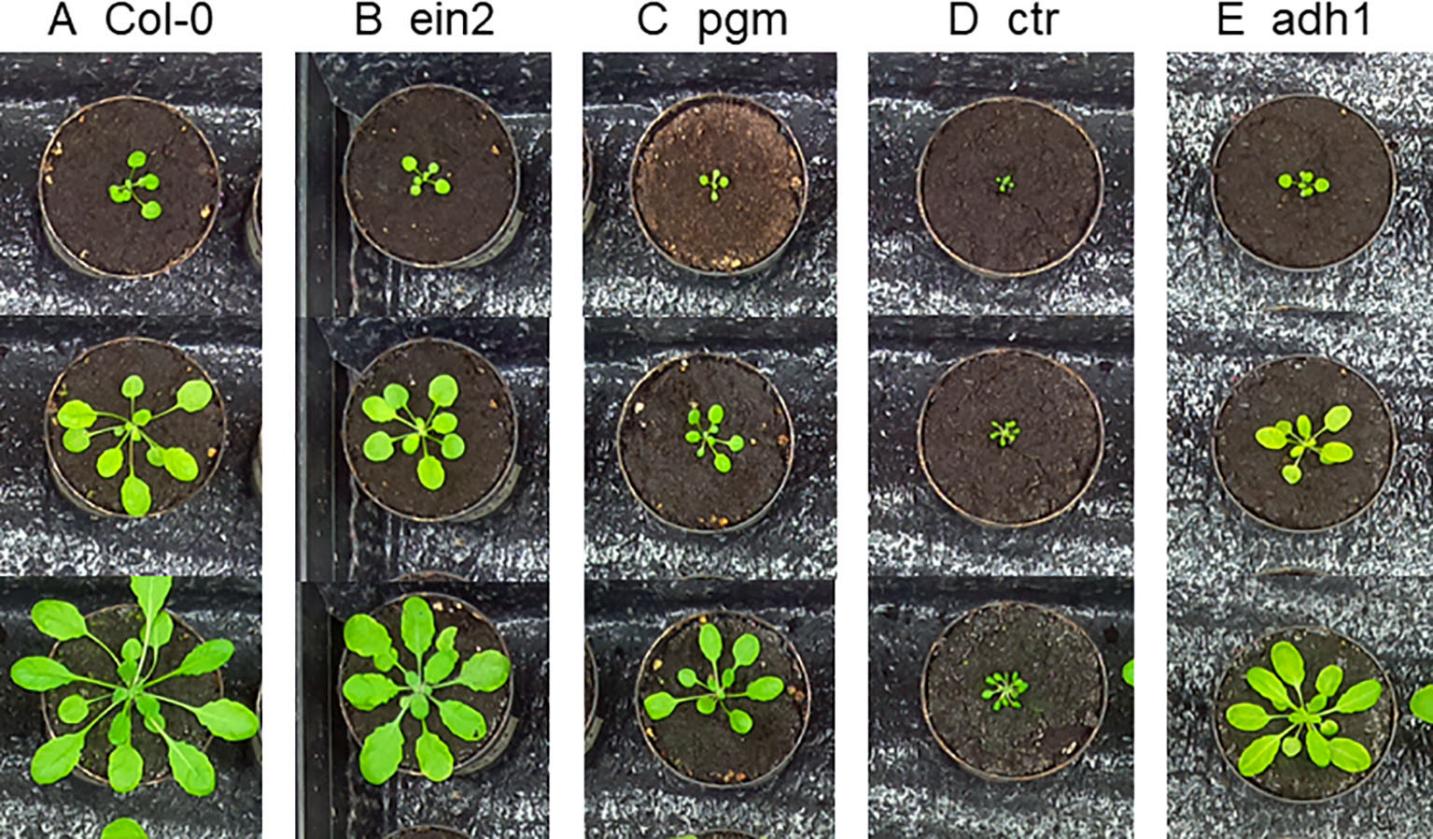
Example images from the *Ara2013* dataset. The dataset is composed of time series images of 24 plants of 5 *Arabidopsis thaliana* accessions. The different genotypes vary in size, shape and color hue, making it a challenging dataset.

Three different datasets were used in this study that contain top-down RGB images of *Arabidopsis thaliana* plants. The *Ara2013* (Minervini et al., 2017) dataset consists of 24 separate plants of 5 different genotypes: Col-0 (wild-type), ein2 (Guzman, 1990), ctr (Kieber et al., 1993), adh1 (Perata and Alpi, 1993), pgm (Caspar et al., 1985). Images were captured of each plant twice a day for 26 days. Example images from the dataset can be seen in **Figure 3**. The different genotypes represent a wide range of visual phenotypes when compared to the wild type (Col-0). Ein2 and adh1 are visually similar to the wild-type while the ctr and pgm are more distinct. Two additional datasets part of the CVPPP leaf counting challenge (LCC), hereafter denoted as A1 (Minervini et al., 2016) and A4 (Bell and Dee, 2016) were also used in evaluating the model. The total number of images in the datasets are 1248, 128, 624 of resolutions 317×309, 500×530, and 441×441 in *Ara2013*, A1, and A4, respectively. The datasets were captured with different experimental setups, so the quality of the images as well as the background appearance varies.

### Data Augmentation

Data augmentation is a method widely used in deep learning to increase the size of available datasets and to give more diverse examples to the neural network during training. The aim is to instill in the model a level of invariance to nuisance factors meaning that the network should give the same results if the same image of a plant is just rotated or shifted. It also helps the network to ignore background variability such as different planting trays, camera setups and soil appearance. For this study, data augmentation was performed when training all models, in the form of assigning random affine transformations from a pool of random rotations between 0 and 180 degrees, shifting the image between 0% and 10% of its size as well as flipping the image on the horizontal or vertical axis.

### Data Preprocessing and Model Training

Before training the neural network, all images were resized to 320 × 320 as a preprocessing step to optimise training times while retaining important features, such as distinct small leaves. Out of the 24 plants in the *Ara2013* dataset, 19 were used for training and the remaining five plants were used for testing. As the five genotypes present in the dataset can be visually distinct, it is important to present the network with an adequate sample of each so that it can learn each genotype particularities. As a result, the five testing plants were chosen as to contain one plant of each genotype. We perform fourfold cross validation where the dataset was randomly divided into four nonoverlapping subsets so that all images are present in the test set once. There is an imbalance between mutants and wild type in the training datasets so a training class weight was added to the genotype classification branch to increase training importance of the wild-type images. The class weight was chosen to be proportional to the class imbalance in each training scenario.

The annotations used during training for each image were the total leaf count as an integer, the PLA, and whether the plant was a mutant or wild type. We normalised the PLA values between 0 and 1 by computing the total area covered by the plant as a percentage of the whole image. In the experiment testing how the model performs with less training annotations, leaf count labels were removed in incremental steps leaving 75%, 50%, and 25% from the total number in the training set. The labels were removed to maintain an even distribution of plant ages and genotypes in the training set (i.e., every 4th label removed for the 75% step). Next, in the experiment analyzing the different strategies of annotating a dataset, three methods of removing labels were employed: we either removed count annotations corresponding to the most juvenile plants, or we removed labels corresponding to the most mature plants, or lastly we removed labels randomly. The same 25% increments were used. During all experiments where we trained the model with fewer leaf count annotations, the PLA and genotype annotations were still provided for all images.

The model was trained on an Nvidia TitanX GPU using the Adam optimizer with a learning rate of 0.0001. All the tasks were concurrently learned end-to-end, with an early stopping criterion based on the validation loss, in order to avoid overfitting. Model selection was according to the overall validation loss for all tasks in the cross-validation.

## Results

In this section, we offer experimental evidence of the effectiveness of our model. To evaluate our model in the leaf counting task, we use CVPPP evaluation metrics (Scharr et al., 2016; Giuffrida et al., 2018b).

They are the difference in count (DiC), absolute DiC (|DiC|), MSE, percentage agreement and coefficient of determination (*R* ^2^). The agreement metric represents the percentage of instances where the network prediction corresponds exactly with the ground truth.

### Evaluation of the MTL Model

**Figure 4:**
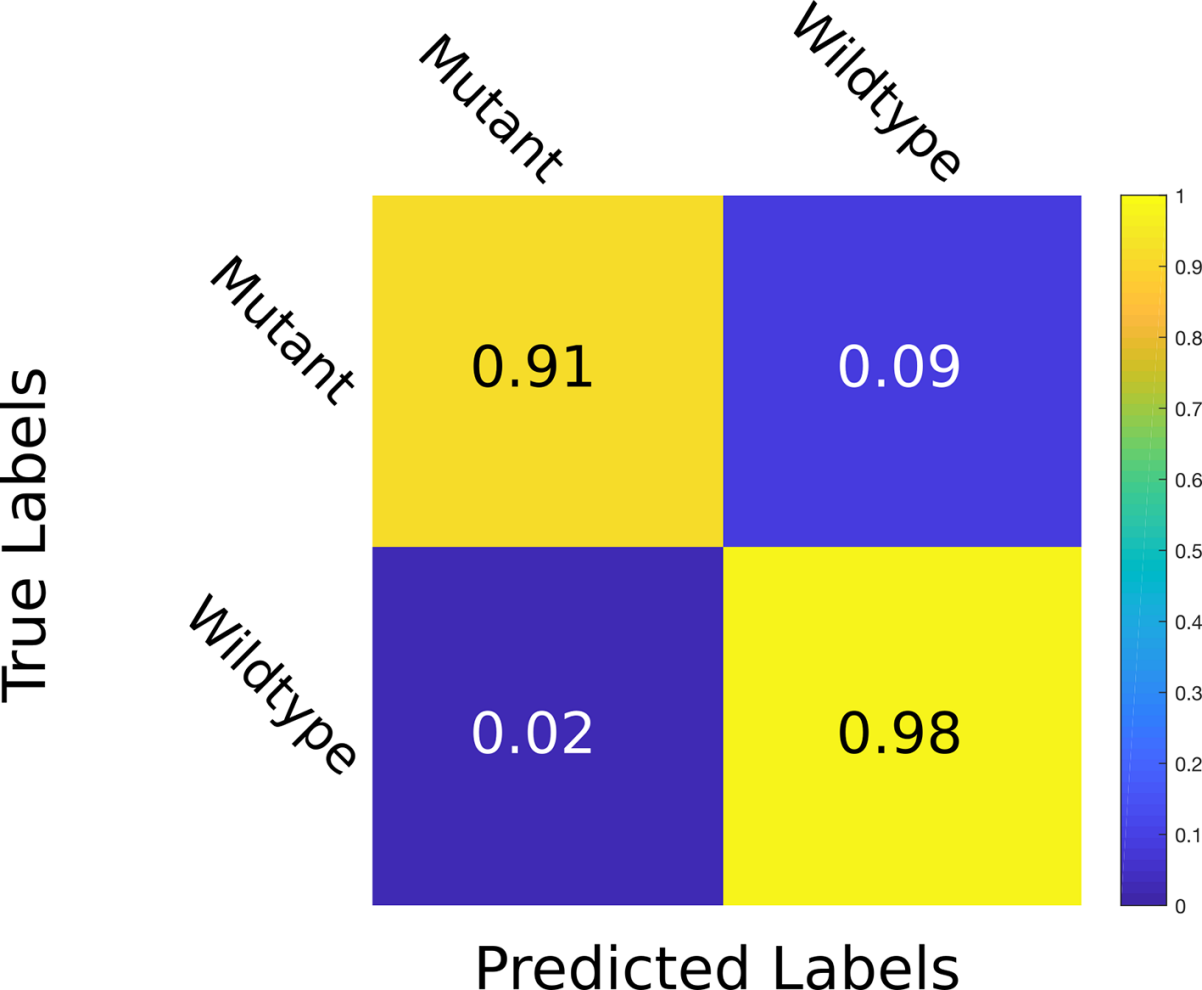
Confusion matrix for genotype prediction at test time. The multitask learning (MTL) model learns to classify whether a plant is a mutant or wild type with an accuracy of 98% correct wild type and 91% correct mutant classification. The values are given at test time in the *Ara2013* dataset.

We first trained our MTL model on the *Ara2013* dataset, as it is the only dataset that contains plants of different genotypes. We then added the A1 and the A4 datasets in order to gauge impact of visually diverse datasets to our model. The results are displayed in **Table 1** for all three tasks. The results show that the network predictions display a strong correlation with the ground truth in the leaf count task with a *R* ^2^ of 0.95 and an overall test MSE of 0.93. The PLA estimation task shows a small MSE equating to an average difference of 2.1% between the ground truth and the predicted PLA. The genotype classification task shows a promising 91.1% test accuracy. As illustrated in the confusion matrix in **Figure 4**, wrong predictions occur rarely. Moreover, the model shows resistance to nuisance variability (i.e., different backgrounds and soil), as we evaluated different datasets grown in different growth scenarios.

**Table 1:** Results for the multitask learning (MTL) network for leaf count and projected leaf area (PLA) and genotype classification.

Dataset	DiC	Count				PLA	Genotype
		│DiC│	Agreement	MSE	R^2^	MSE	Accuracy
*Ara2013*	−0.22 (0.93)	0.67 (0.69)	45	0.93	0.95	0.021	91.1
*Ara2013* + *A1A4*	−0.21 (1.09)	0.77 (0.79)	44	1.23	0.96	0.025	95.6

The values are computed at test time for the model trained on first just the *Ara2013* dataset and then the extended dataset of *Ara2013*+A1+A4. The small drop in performance in the extended dataset can be attributed to the increase in dataset difficulty by adding more challenging examples.

Next, we assessed if the addition of MTL increases performance for the leaf counting task compared to a single task model. To make the single task leaf count variant of our model we removed the other branches. We maintained the same training procedure for both models and the dataset used was the *Ara2013* dataset. The results are shown in **Table 2**. Overall, the results of the MTL model are improved for all metrics analyzed, demonstrating that MTL reduces prediction errors when multiple related tasks are learned jointly. To test whether there is a statistically significant difference between the multitask and single-task models, we performed a bootstrapped paired t-test (Rodriguez, 2011) between the results of the |DiC| for the two approaches at 100% leaf count labels with a null hypothesis that they are equal. We perform the bootstrapped t-test because the output of our model for the |DiC| does not follow a Gaussian distribution required for a valid t-test. The result is a two tailed p-value of 0.0093 after 10^5^ bootstrapped samples. The p-value of <0.05 demonstrates that there is a significant difference between the MTL and single task models, confirming the superiority of MTL for the leaf counting task.

**Table 2:** Results for the multitask learning (MTL) network vs. the single task network for leaf counting task trained on the *Ara2013* dataset.

Model	DiC	│DiC│	Agreement	MSE	R^2^
Single task	0.40 (1.09)	0.80 (0.84)	41	1.35	0.92
Multitask	0.22 (0.93)	0.67 (0.69)	45	0.93	0.95

All parameters are improved in the MTL model, with the mean squared error (MSE) showing an improvement of 40%.

We then compare our MTL framework to current state of the art specialised leaf counting models. The results can be seen in **Table 3**. We trained our MTL model on the A1 dataset but with just the leaf count and PLA tasks as there are only wild-type plants present. Our model outperforms the results of Dobrescu et al. (2017a) in all categories. We achieve similar results to the best reported values in Itzhaky et al. (2018), D+R method that utilise the leaf center as additional training annotation, while we are using only a direct regression method.

**Table 3:** Comparison of our proposed multitask learning (MTL) model with state-of-the-art results in leaf counting on the *Computer Vision Problems in Plant Phenotyping* workshop (CVPPP) A1 test set.

Method	DiC	│DiC│	Agreement	MSE
Romera-Paredes and Torr (2016)**	0.20(1.40)	1.1(0.9)	–	–
Aich and Stavness (2017) ^†^	−0.33(1.38)	1.00(1.00)	30.3	1.97
Dobrescu et al. (2017a) ^†^	−0.39(1.17)	0.88(0.86)	33.3	1.48
Itzhaky et al. (2018) MSR^†^	−0.27(1.21)	0.70(1.02)	57.0	1.48
Itzhaky et al. (2018) D+R^**^	−0.12(1.11)	0.73(0.84)	45.5	1.21
Proposed Multi-Task Model^†^	−0.09(1.10)	0.78(0.77)	39.0	1.22

The results show an improvement in mean squared error (MSE) on previous works that use just the total leaf count as annotation. The results are similar to the current state-of-the-art specialized leaf counting networks. The table only shows results of the leaf counting task as there is no benchmark for the other tasks. ^†^Method uses just the total leaf count as annotation. ^**^Method uses stronger annotations.

### Substituting Hard to Get Annotations With MTL

In this experiment, we assess whether we could compensate for the lack of expensive training annotations in the leaf counting task by using an MTL approach and providing other, easier to acquire, annotations. When training the network, we removed parts of the leaf count labels, but we retained all the PLA and genotype labels. Leaf count labels were removed in incremental steps leaving 75%, 50%, and 25% from the total number in the training set to check how the models perform when increasingly fewer count annotations are available.

Experimental results are shown in **Table 4**. It can be noted that the MTL model remain consistent even when only 25% of the original count labels are used in training. Furthermore, the standard deviation of the DiC in the MTL model remains nearly constant for all the label steps, indicating that the predictions are consistently close to the reported mean. On the other hand, the single-task model sees a significant decline in performance when less training annotations are present. The MSE increases from 1.45 when 100% of the labels are present to 5.49 and then to 17.2 at the 50% and 25% count label steps respectively.

**Table 4:** Effect of incrementally decreasing leaf count annotations in the multitask learning (MTL) (multi) and single-task (single) models during training.

	Count Labels	100%	75%	50%	25%
DiC	Single Multi	0.40 (1.09) −0.22 (0.93)	0.82 (1.68) −0.14 (0.94)	1.16 (2.04) −0.23 (0.95)	1.18 (3.98) −0.46 (0.94)
│DiC│	Single Multi	0.80 (0.84) 0.67 (*0.69)	1.28 (1.36) 0.62 (0.71)	1.62 (1.69) 0.75 (0.72)	2.68 (3.16) 0.75 (0.75)
Agreement	Single Multi	41 45	33 48	23 40	21 42
MSE	Single Multi	1.35 0.93	3.48 0.91	5.50 1.08	17.2 1.13
R^2^	Single Multi	0.92 0.95	0.80 0.95	0.68 0.94	0.02 0.94

All the projected leaf area (PLA) and genotype labels are still present during training of the MTL model. The MTL model maintains steady performance in all label steps while the single task model shows significant decline. We show the results on the leaf count task because it is the most challenging task.

The same trend is visible in the *R* ^2^ values as well declining from 0.92 at 100% to near 0 when only 25% of the count labels are available. To test whether there is a significant difference between the results of the different count label thresholds in **Table 4**, we computed the same type of bootstrapped paired sample t-tests mentioned in Section 3.1 between the results of the |DiC| for the multitask and single-task models trained with 100% and 25% of the labels respectively, using the standard threshold of 0.05 as a significance level to indicate whether there is a true mean difference between the two samples. The performance drop is more noticeable in single task-model at all levels of omitted labels and the bootstrapped two tailed p-value well below <0.001 reflects the results. On the other hand, in the MTL model, the results remain stable and do not differ significantly as the number of training labels decrease (bootstrapped two tailed p-value of 0.097, above significance threshold). This means that the model successfully compensates from the lack of leaf counting data by learning from the other tasks.

**Figure 5:**
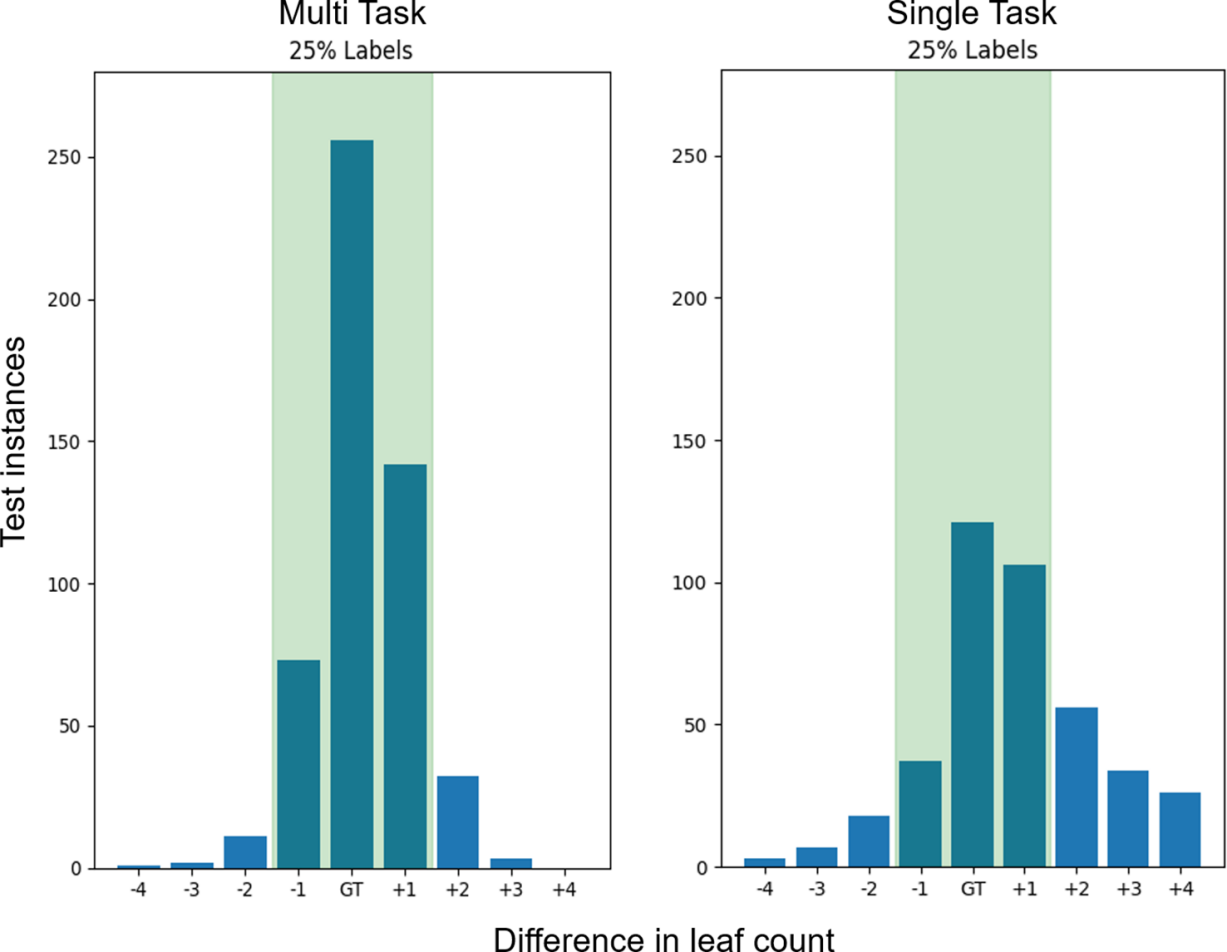
Leaf count test accuracy when training with 25% of the count labels. The bars represent the sum of predictions in the *Ara2013* test set which are equal the ground truth as well as the differences in count on either side. The green shaded region represents the region of ground truth ±1 leaves which is similar to human accuracy. The multitask learning (MTL) variants have a ±1 accuracy of 91% while the single task models only have a 50% ± 1 accuracy and a much wider spread of difference in count errors.

The distribution of count predictions at the 25% count label step can be seen in **Figure 5**. The MTL model maintains a more leptokurtic distribution, with 91% of the predictions fall within ±1 of the ground truth, compared to the single task model where only 50% of predictions are within ±1 of the ground truth.

### Which Labels Are Most Important

Given that it is possible to obtain reliable leaf count predictions with only the 25% of the training count labels in the MTL model, an important question is: *Which 25% of labels are most important for the model to successfully train?* Understanding this key aspect would help guide the first annotation of a new dataset, highlighting which 25% of images should be first (and potentially only) annotated in a new dataset.

We evaluated three different annotating strategies and results are shown in **Table 5**. Firstly, we removed the count labels starting with the youngest plants up to the designated thresholds of 75%, 50%, and 25%. For example, at 50% labels there were no count labels present for the first half of the plant’s life. Using this method, the results show similar results between 100% and 75% count labels so we conclude that the youngest plants have little impact on the training of the model. The results then start declining until there is only a *R* ^2^ value of 0.07 when only the oldest 25% of the plants were present.

**Table 5:** The impact on the multitask learning (MTL) model different strategies for annotating a dataset by determining the impact on the MTL model count labels and their impact on the MTL model.

			Count			PLA	Genotype
Selection Method	Count Labels	│DiC│	Agreement	MSE	R^2^	MSE	Accuracy
All count labels	100%	0.67 (0.69)	45	0.93	0.95	0.021	90
Removed juvenile plants	75% 50% 25%	0.65 (0.68) 1.66 (1.73) 3.45 (2.26)	46 28 6	0.89 5.76 17.08	0.95 0.68 0.07	0.025 0.032 0.030	91 88 81
Removed mature plants	75% 50% 25%	1.36 (1.84) 4.83 (5.80) 6.91 (6.20)	34 21 17	5.27 53.03 86.16	0.71 N/A N/A	0.015 0.013 0.019	63 73 67
Random Selection	75% 50% 25%	0.70 (0.68) 0.67 (0.71) 1.39 (1.24)	42 44 27	0.97 0.96 3.49	0.94 0.94 0.81	0.010 0.024 0.045	91 88 81

This can also be seen as what count labels are most important when annotating a new image based plant growth dataset. The values shown were obtained training on the *Ara2013* dataset. The count labels were removed in increments of 25%. First the labels of the most juvenile plants were removed. Then the labels of the oldest plants were removed. The third category removes count labels in a random fashion at the designated percentage steps.

The next method is the reverse of the previous one, meaning we removed the count labels starting with the oldest plants. We observed a decline in results, even at 75% labels. At the next step threshold, the model failed to learn any of the tasks. Lastly, we excluded annotations from the dataset selecting at random plants across the time span. This method, perhaps as expected, gave results which are comparable to having an equal distribution of labels as in **Table 4**. At the 25% step the results worsened, but this could be explained due to random chance of how the count label distribution was selected.

### Determining Important Image Regions

Training a deep neural network model with less annotations generally makes it more difficult for the model to learn. To assess this impact in our model, we investigated what parts of the image the network considers important. We aim to see if the most important regions correspond to the plant or the network is influenced by information found in the background (e.g. the soil or plant pot). We performed the test by imposing a black sliding window on a sample of test images and predicted the leaf count, genotype, and PLA using our model on the images as the sliding window was traversing it. The method developed in Zeiler and Fergus (2014) is similarly used in Dobrescu et al. (2017a). The aim was to understand what are the important parts of the image from the trained network’;s perspective, as obstructing such a part would give rise to errors in the predictions.

**Figure 6:**
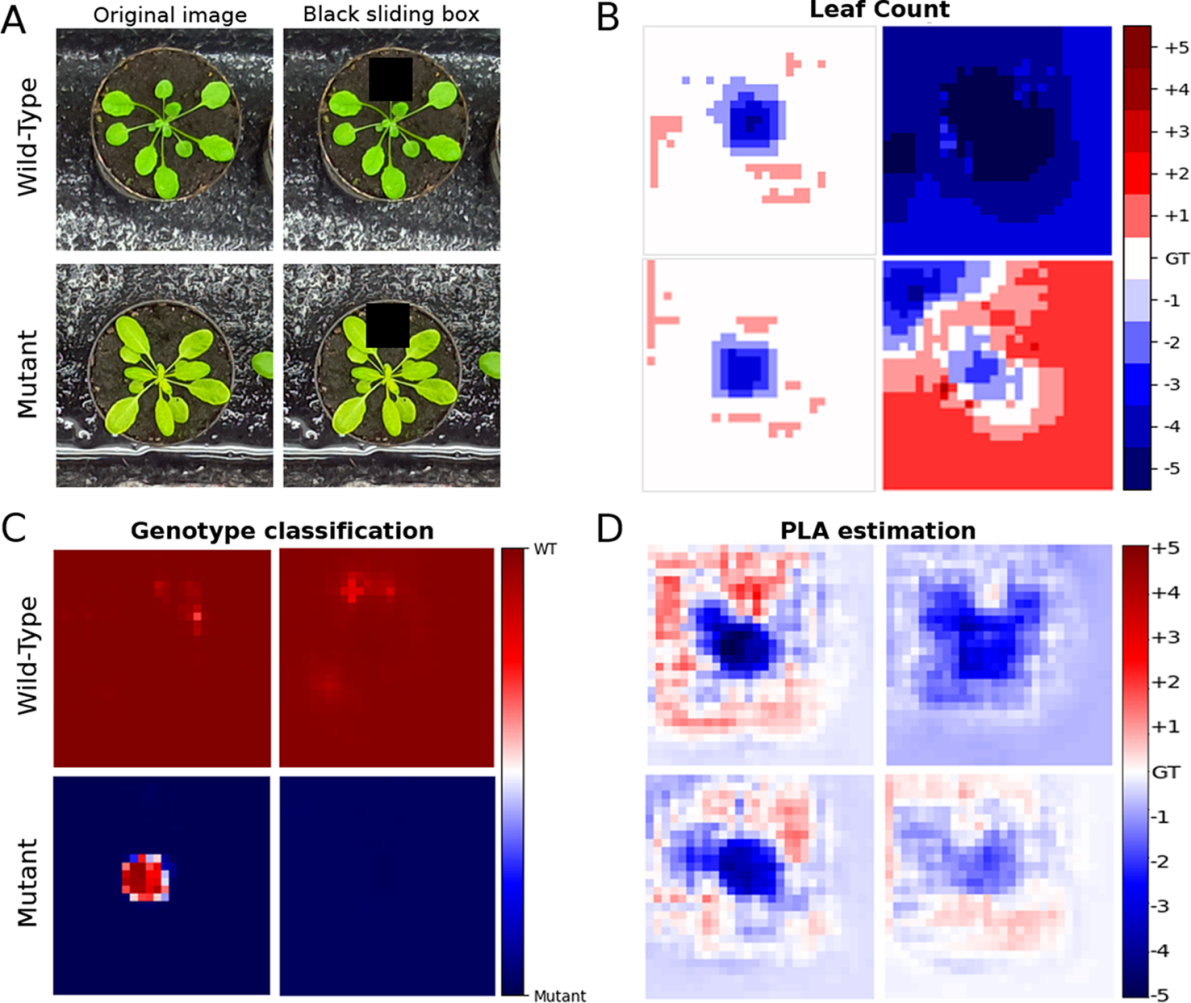
Test showing the focus of the network using a sliding window. **(A)** is the original test image, along with the count ground truth. Overlaid is a black sliding window (60 × 60 pixels), which traverses the original images. The top row is an example of a wild type plant and the bottom row is an example of a mutant. **(B)** represents the prediction accuracy as the sliding window is traversing the image of the multitask learning (MTL) model (left column) and the single task model (right column) trained with just 25% count labels. The errors are expected to be confined to the area where the plant is located as the box obscures whole or parts of leaves the overall count prediction should decrease. **(C**, **D)** represent the sliding box test only in the MTL model for Genotype classification accuracy and projected leaf area (PLA) estimation. These were only performed on the MTL model comparing between models trained with 100% (right column) and 25% (left column) labels. The color bar for the PLA task shows increments in percentage points. The rows correspond to the images in part A. GT signifies the ground truth.

For the leaf counting task, we carried out this test on models trained using 25% count annotations in MTL and single task models to gauge if there is a difference in how the errors are distributed in the two approaches when less annotations were available. In the PLA estimation and genotype classification we compare MTL models trained with 100% and 25% count labels to determine if they were learning properly, and if they were still focusing on relevant image parts at the two annotation increments. The results are shown in **Figure 6**, showing that the network does actually focus mostly on the image areas corresponding to the plants. Additionally in the MTL model the errors generated and the regions impacted are similar between the models trained with 100% and 25% leaf count labels.

## Discussion

We show that an MTL deep learning approach is superior to just single task models for the purposes of characterizing visually challenging plant traits, such as leaf counting. We treat the leaf counting problem as a holistic regression task. One of the limitations of such approaches is that the network needs to learn good image representation from each image, based only on the total leaf count number. Employing an MTL model offers extra information to the model easing the training process.

In agreement with Caruana (1997), an MTL model can learn also from the other tasks leading to better generalization performance and more robust extraction of features. The benefits can be seen in **Tables 2** and **4** where the MTL model outperforms the single-task model. The performance of deep neural networks is known to be strongly influenced by the quantity of annotated data used during training (Sun et al., 2017). By omitting leaf count labels in our approach, the model is essentially training the leaf counting task with fewer annotated examples and, therefore, it would be expected to have an important negative impact on the results. However that is not the case for our MTL model, which can overcome the extra difficulty of training from less annotations without having a statistically significant drop in performance. Furthermore, during training, the MTL model was more stable when fewer count annotations were available compared to the single task models (see Additional **Figure 1** for more details).

Getting a sense of what regions the network considers important, provides an insight if the model was successfully trained to get information from the appropriate image areas (i.e., the plant not the background). There is a clear difference between the MTL and single task models in the leaf counting task when trained with just 25% count annotations **Figure 6**. As the sliding box moves over the image, the errors that produce a lower count prediction are very specific to regions containing the plant suggesting that the model learned well the area of interest. On the other hand, the single task model yields more pronounced prediction errors in all regions of the image so it does not focus on the plant region as well as the MTL model. For the genotype classification and PLA estimation tasks we investigated if there are differences between MTL models trained with 100% and 25% leaf count annotations. There is no visibly significant difference between them meaning that both models learned to focus mostly on the plant areas.

Two of the mutants present in the *Ara2013* dataset seen in **Figure 3**, (ein2 and adh1) are visually similar to the Col-0 wild type, making genotype classification a challenging task. The errors we observed occur mainly when the model misclassifies these mutants as wild-type in the early and middle part of the growth cycle. However, the overall classification accuracy remains >90%, demonstrating that the network is not biased towards a specific class.

When assessing the best strategy to select labeled data for the leaf counting task in the MTL model, we can find what are the most important time points in the plant growth stage for the network to learn in **Table 5**. The network performance is directly affected when the count labels are missing from mature plants, while minor changes are seen when the juvenile 25% are removed, showing similar behavior as a random selection of up to 50%. This means that most important information for these tasks is learned from the mature plants, while the juvenile plants contribute less in the learning process. The other tasks reflect this trend as well. We can conclude that the best strategy is to provide the most balanced dataset, that provides the widest-ranging examples to the neural network during training. Next, in order from best to worst would be to just randomly choose which labels to provide, then omitting the juvenile plants and lastly is to omit the mature ones.

## Conclusions

In this paper we have proposed a framework for multitask deep learning (MTL) for plant phenotyping. We showed that MTL architecture outperforms the single-task models trained on the same datasets. We have achieved an improvement on the state-of-the-art for leaf counting compared to direct regression approaches for the datasets tested. We achieve a similar performance to state-of-the-art methods which use additional annotations for training. To the best of our knowledge, this is the first work that studies and compares the benefits of MTL versus single task in plant phenotyping. We show that the proposed MTL model can be used to compensate for missing labels in plant phenotyping, leveraging other related traits. We have also explored different leaf count annotation strategies and showed which segments of the plant images are most important to be labeled. Lastly we have shown that the MTL model correctly focuses on the parts of the image that correspond to the plant and largely disregards the background when computing prediction for all three tasks.

## Data Availability Statement

Publicly available datasets were analyzed in this study. This data can be found here: https://www.plant-phenotyping.org/CVPPP2017-challenge.

## Author Contributions

AD, MG, and ST conceived the study. AD performed the experiments. AD prepared the manuscript with feedback from MG and ST. All authors read and approved the final manuscript.

## Funding

AD is currently supported by an EPSRC DTP PhD fellowship (EP/N509644/1). MG is supported by the BBSRC grant BB/P023487/1. ST is partly supported by the UK Biotechnology and Biological Sciences Research Council (BB/P023487/1) and The Alan Turing Institute under the EPSRC grant EP/N510129/1.

## Conflict of Interest

The authors declare that the research was conducted in the absence of any commercial or financial relationships that could be construed as a potential conflict of interest.

## Footnotes

1 More information available at https://www.plant-phenotyping.org/CVPPP2019
